# Analysis of Clinical Manifestations and Prognostic Factors Affecting Osteosarcoma

**DOI:** 10.1155/2022/1599112

**Published:** 2022-08-04

**Authors:** Jianghua Wei, Zhizhong Liang, Gang Xu

**Affiliations:** Department of Bone and Soft Tissue Oncology, Shanxi Provincial Cancer Hospital, Taiyuan, China

## Abstract

**Objective:**

This study is aimed at exploring the clinical manifestations and prognostic factors of osteosarcoma.

**Methods:**

The clinical data of patients with osteosarcoma who were treated in our hospital from January 2018 to March 2020 were selected for retrospective analysis. The general information of the patients, including age, gender, tumor diameter, tumor location, tumor type, surgical method, and Enneking stage, distant disease metastasis, KPS score, and the number of postoperative adjuvant chemotherapy, were grouped by prognosis for statistical analysis. The clinical characteristics, morbidity and mortality, and prognostic factors of patients were statistically analyzed.

**Results:**

Among the 83 patients in this group, there were 52 males and 31 females, 59 tumors > 10 cm in diameter and 24 tumors < 10 cm in diameter, 16 tumors in the upper limbs and 67 tumors in the lower limbs, 25 tumors in osteoblastoma, 16 tumors in chondroblastoma, 42 tumors in fibroblastoma, 62 tumors in stage II, and 21 tumors in stage III of Enneking stage, 10 tumors in distant metastasis, and 10 tumors in distant metastasis. The death rate of this group was 19.28% (16/83). Multifactor regression analysis confirmed that the Enneking stage III, distant metastasis, KPS score < 70, and the number of postoperative adjuvant chemotherapy < 6 were important factors influencing the death of osteosarcoma (*P* < 0.05).

**Conclusion:**

Enneking stage III, distant metastasis, KPS score < 70, and the number of postoperative adjuvant chemotherapy < 6 are important influencing factors of osteosarcoma death. Clinical practice can take corresponding preventive and control measures according to the existence of these factors to ensure a good prognosis.

## 1. Introduction

Osteosarcoma is a type of malignant bone tumor with a high incidence of malignancy, accounting for 15% of the total incidence of bone tumors. It is highly malignant, has a complex pathogenesis and progression, and can develop at any age [1] . Osteosarcoma has a high mortality rate and a poor prognosis, posing a great threat to the quality of life and physical and mental health of patients [2] . Surgery is an important treatment for osteosarcoma and has been used in the past for high/superarticular amputation, but it is very likely to affect the quality of life due to disability and has a low 1-year survival rate [3] . With the maturation of adjuvant interventional techniques such as chemotherapy, limb preservation has been widely used, but there is still a certain risk of death after limb preservation [4]; therefore, it is important to clarify the clinical manifestations and prognostic factors of osteosarcoma patients. This study is aimed at exploring the clinical manifestations and prognostic factors of osteosarcoma.

## 2. Data and Methods

### 2.1. General Information

In this study, the clinical data of patients with osteosarcoma who were treated in our hospital from January 2016 to March 2020 were selected for retrospective analysis. The inclusion criteria for this study were as follows: (1) confirmed by pathology and imaging, (2) osteosarcoma of the extremities, and (3) complete clinical data. The exclusion criteria for this study are as follows: (1) patients with metastases at the time of consultation, (2) patients with a history of tumor boundary destruction treatment, (3) patients with other systemic malignant tumors, (4) patients with other serious diseases, such as heart disease or diabetes, (5) those who died of other reasons during the study period, (6) those who are pregnant or breastfeeding, and (7) those who have diseases of the immune system and blood system.

### 2.2. Methodology

Patients were grouped according to their prognosis, and the general conditions of all patients were statistically analyzed, including age, gender, tumor diameter, tumor location, tumor type, surgical method, Enneking stage, distant metastasis of the lesion, KPS score, and number of postoperative adjuvant chemotherapy [5, 6]. Statistical analysis was performed by two specially trained physicians, and when the results of the two were inconsistent, a third physician performed reanalysis.

### 2.3. Observed Indicators

Here are the following examples of the observed indicators: statistical analysis of the clinical characteristics of the patients in this group, statistical analysis of the morbidity and mortality rate of the patients in this group, and statistical analysis of the factors influencing the prognosis of osteosarcoma.

### 2.4. Statistical Analysis

The data were analyzed by using SPSS 22.0 software (IBM Statistics, Chicago, USA), the count data were presented as case number using the *χ*^2^ test, the measurement data were confirmed to have chi-squared and to be approximately normally distributed by the Bartlett chi-squared test and the Kolmogorov-Smirnov normality test and were described by the mean ± standard deviation (SD), and the factors influencing the prognosis of osteosarcoma were analyzed by logistic multiple. The factors influencing the prognosis of osteosarcoma were analyzed by logistic regression. *P* value less than 0.05 was considered as significant difference.

## 3. Results

### 3.1. Analysis of Clinical Characteristics of This Group of Patients

This retrospective analysis contained 83 patients. The tumor types were osteoblastoma in 25 cases, chondroblastoma in 16 cases, fibroblastoma in 42 cases, stage II in 62 cases, and stage III in 21 cases. There were 10 cases with distant metastases and 73 cases without metastases.

### 3.2. Analysis of the Morbidity and Mortality Rate of Patients in This Group

Of the 83 patients in this group, 16 died of disease and 67 survived, with a morbidity and mortality rate of 19.28% (16/83).

### 3.3. Univariate Analysis of Factors Influencing Prognosis of Osteosarcoma

As shown in Table 1, there was no statistically significant difference in the survival of patients with osteosarcoma with different ages, gender, tumor diameter, tumor location, tumor type, and surgical methods (*P* > 0.05), and there was a statistically significant difference in the survival of patients with osteosarcoma with different Enneking stage, distant metastasis of the lesion, KPS score, and number of postoperative adjuvant chemotherapy (*P* < 0.05).

### 3.4. Multifactorial Analysis of Factors Affecting Prognosis of Osteosarcoma

With osteosarcoma death as the dependent variable and Enneking stage, distant metastasis of the lesion, KPS score, and number of postoperative adjuvant chemotherapy as the independent variables, it was confirmed by multifactorial regression analysis that Enneking stage III, distant metastasis of the lesion, KPS score < 70, and number of postoperative adjuvant chemotherapy < 6 were significant influencing factors for osteosarcoma death (*P* < 0.05, Table 2).

## 4. Discussion

Osteosarcoma is a type of primary malignant tumor of bone that occurs in children and adolescents, with a high degree of malignancy and a high rate of disability and death [7, 8]. In recent years, with the improvement of disease treatment technology, the survival rate of osteosarcoma has improved, but the overall prognosis is still difficult to reach the expected clinical level [9, 10]. Therefore, the disease characteristics and prognostic factors should be clarified, so that corresponding interventions can be made to minimize the risk of death.

This study retrospectively analyzed the clinical data of patients with osteosarcoma in our hospital. Our results show that Enneking stage III, distant metastasis, KPS score < 70, and the number of postoperative adjuvant chemotherapy < 6 are important factors that affect the death of osteosarcoma. The above factors may affect the death of osteosarcoma. The prognosis of patients with osteosarcoma has a significant impact because the Enneking stage is significantly positively correlated with the number of primary lesions, disease severity, and onset speed of disease progression. The Enneking stage was significantly positively correlated with the malignancy of osteosarcoma lesions, the severity of the disease, and the rate of disease progression, and the stage of the disease not only predicted the number of primary lesions but also the number of potential metastatic lesions. The KPS score can reflect the physical and functional status of patients with osteosarcoma. The lower the KPS score, the poorer the physical function, and it is usually difficult to tolerate radiotherapy treatment, which will affect the frequency and dose of radiotherapy and chemotherapy. Such patients are usually difficult to complete standard radiotherapy treatment. The prognosis is poor. Surgery can remove primary osteosarcoma lesions, but distant metastases are difficult to remove, which is also the main factor for the recurrence of osteosarcoma after treatment. Distant proliferation and metastasis of lesion cells can cause the disease. Postoperative adjuvant chemotherapy for osteosarcoma can excise distal metastatic osteosarcoma lesions or clear the lesions under microscope, thereby prolonging the survival period [11–13]. Therefore, when treating osteosarcoma, effective nutritional support should be given to those who have factors that affect the good outcome of the disease, so as to improve their body condition, enhance their chemotherapy tolerance, and ensure complete and standardized adjuvant chemotherapy treatment after surgery, thus completely eliminating lesions and metastases, prolonging the survival cycle, and improving the prognosis [14, 15]. Another study confirmed that postoperative pathological fracture is also an important factor affecting the prognosis of osteosarcoma, mainly because the pathological feature of osteosarcoma is osteolytic bone damage, and the higher the malignancy and longer the disease duration, the higher the bone fragility and the higher the risk of pathological fracture; therefore, pathological fracture patients usually have higher disease stage, higher malignancy, and longer disease duration; so, the risk of good regression is more difficult [16, 17], which is consistent with the conclusion of this study. There is a certain consistency with the findings of this study, and the clinical practice can promote good disease regression through comprehensive interventions such as enhancing bone density and nutrition.

In summary, Enneking stage III, distant metastasis of the lesion, KPS score < 70, and the number of postoperative adjuvant chemotherapy < 6 are important factors influencing the death of osteosarcoma, and the clinical practice can take corresponding preventive and control measures according to the presence of the above factors to ensure the prognostic effect of the disease.

## Figures and Tables

**Table 1:** Univariate analysis of factors influencing prognosis of osteosarcoma.

Entry		Number of cases	Survival (*n* = 67)	Sickness and death (*n* = 16)	*χ* ^2^	*P*
Gender	Male	52	42 (62.69)	11 (68.75)	0.206	0.650
	Female	31	25 (37.31)	5 (31.25)		
Age	<18 years old	44	36 (53.73)	8 (50.00)	0.072	0.788
	≥18 years old	39	31 (46.27)	8 (50.00)		
Tumor diameter	≥10 cm	59	48 (71.64)	11 (68.75)	0.053	0.819
	<10 cm	24	19 (28.36)	5 (31.25)		
Tumor location	Upper extremity	16	13 (19.40)	3 (18.75)	0.086	0.769
	Lower extremities	67	54 (80.60)	13 (81.25)		
Tumor type	Osteoblastoma	25	20 (29.85)	5 (31.25)	0.013	0.994
	Chondroblastoma	16	13 (19.40)	3 (18.75)		
	Fibroblastoma	42	34 (50.75)	8 (50.00)		
Surgical method	Limb preservation surgery	44	35 (52.24)	9 (56.25)	0.083	0.773
	Amputation surgery	39	32 (47.76)	7 (43.75)		
Enneking installment	Period II	62	61 (91.04)	1 (6.25)	49.136	0.001
	III period	21	6 (8.96)	15 (93.75)		
Distant metastasis of the lesion	Yes	10	4 (5.97)	6 (37.50)	12.117	0.001
	No	73	63 (94.03)	10 (62.50)		
KPS score	≥70 points	55	48 (71.64)	7 (43.75)	4.495	0.034
	<70 points	28	19 (28.36)	9 (56.25)		
Number of adjuvant chemotherapy after surgery	≥6 times	68	63 (94.03)	5 (31.25)	34.381	0.001
	<6 times	15	4 (5.97)	11 (68.75)		

**Table 2:** Multifactorial analysis of factors influencing prognosis of osteosarcoma.

Variables	*β*	S.E.	Wald *χ*^2^	*P*	OR	95% CI
Enneking staged as stage III	1.189	0.405	8.621	<0.001	3.284	1.776~6.073
Distant metastasis of the lesion	1.340	0.461	8.451	<0.001	3.820	2.044~7.138
KPS score < 70 points	1.578	0.535	8.698	<0.001	4.845	3.665~6.404
Number of postoperative adjuvant chemotherapy < 6 times	1.698	0.601	7.984	<0.001	5.464	4.098~7.286

## Data Availability

The data was within our manuscript.

## References

[1] Ding W. Z., Liu K., Li Z., Chen S. R. (2020). A meta-analysis of prognostic factors of osteosarcoma. *European Review for Medical and Pharmacological Sciences*.

[2] Sun H. H., Chen X. Y., Cui J. Q., Zhou Z. M., Guo K. J. (2018). Prognostic factors to survival of patients with chondroblastic osteosarcoma. *Medicine (Baltimore)*.

[3] Xu G., Wu H., Xu Y. (2021). Homogenous and Heterogenous prognostic factors for patients with bone sarcoma. *Orthopaedic Surgery*.

[4] Testa S., Hu B. D., Saadeh N. L. (2021). A retrospective comparative analysis of outcomes and prognostic factors in adult and pediatric patients with osteosarcoma. *Current Oncology*.

[5] Meng T., Yin H., Li B. (2015). Clinical features and prognostic factors of patients with chordoma in the spine: a retrospective analysis of 153 patients in a single center. *Neuro-Oncology*.

[6] Xu M., Wang Z., Yu X. C., Lin J. H., Hu Y. C. (2020). Guideline for limb-salvage treatment of osteosarcoma. *Orthopaedic Surgery*.

[7] Abou Ali B., Salman M., Ghanem K. M. (2019). Clinical prognostic factors and outcome in pediatric osteosarcoma: effect of delay in local control and degree of necrosis in a multidisciplinary setting in Lebanon. *Oncologia*.

[8] Zhang C., Guo X., Xu Y. (2019). Lung metastases at the initial diagnosis of high-grade osteosarcoma: prevalence, risk factors and prognostic factors. A large population-based cohort study. *São Paulo Medical Journal*.

[9] Yamamoto Y., Kanzaki R., Kanou T. (2019). Long-term outcomes and prognostic factors of pulmonary metastasectomy for osteosarcoma and soft tissue sarcoma. *Journal of Clinical Oncology*.

[10] Kim W., Han I., Lee J. S., Cho H. S., Park J. W., Kim H. S. (2018). Postmetastasis survival in high-grade extremity osteosarcoma: a retrospective analysis of prognostic factors in 126 patients. *Journal of Surgical Oncology*.

[11] Cates J. M. M. (2018). Modeling continuous prognostic factors in survival analysis. *The American Journal of Surgical Pathology*.

[12] Bosma S. E., Ayu O., Fiocco M., Gelderblom H., Dijkstra P. D. S. (2018). Prognostic factors for survival in Ewing sarcoma: a systematic review. *Surgical Oncology*.

[13] Jawad M. U., Bayne C. O., Farhan S. (2021). Prognostic factors, disparity, and equity variables impacting prognosis in bone sarcomas of the hand: SEER database review. *Journal of Surgical Oncology*.

[14] Liu W. F., Huang Z., Gong L. H. (2019). Synchronous multicentric osteosarcoma: treatment and prognostic factor analysis. *Natl Med J China*.

[15] Alvarez-SanNicolas J., Gracia-Alegria I., Trullols-Tarrago L., Peiro-Ibañez A., Lamas-Gomez C. (2019). Prognostic factors and survival in Ewing's sarcoma treated by limb salvage surgery. *Clinical & Translational Oncology*.

[16] Restrepo D. J., Huayllani M. T., Boczar D. (2019). Which factors affect survival in patients with upper limb osteosarcoma?. *Anticancer Research*.

[17] Imura Y., Takenaka S., Kakunaga S. (2019). Survival analysis of elderly patients with osteosarcoma. *International Orthopaedics*.

